# AC Magnetometry Using Nano-ferrofluid Cladded Multimode Interferometric Fiber Optic Sensors for Power Grid Monitoring Applications

**DOI:** 10.1021/acsanm.4c04912

**Published:** 2024-11-23

**Authors:** Dolendra Karki, Tulika Khanikar, Suraj V. Mullurkara, Khurram Naeem, Jun Young Hong, Paul Ohodnicki

**Affiliations:** † Mechanical Engineering & Materials Science, University of Pittsburgh, Pittsburgh, Pennsylvania 15261, United States; ‡ Electrical and Computer Engineering, University of Pittsburgh, Pittsburgh, Pennsylvania 15261, United States

**Keywords:** AC magnetometry, optical-fiber current sensor, nano-ferrofluid, magnetic nanoparticles, multimode interferometry, current faults detection and monitoring

## Abstract

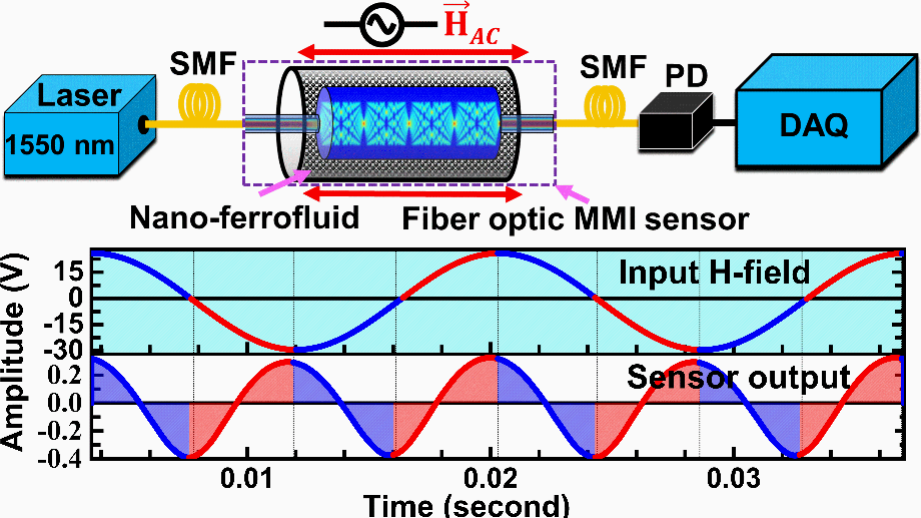

The AC magnetic field response of the superparamagnetic nano-ferrofluid is an interplay between the Neel and Brownian relaxation processes and is generally quantified via the susceptibility measurements at high frequencies. The high frequency limit is dictated by these relaxation times which need to be shorter than the time scale of the time varying magnetic field for the nano-ferrofluid to be considered in an equilibrium state at each time instant. Even though the high frequency response of ferrofluid has been extensively investigated for frequencies up to GHz range by non-optical methods, harnessing dynamic response by optical means for AC magnetic field sensing in fiber-optic-based sensors-field remains unexplored. Instead, the incorporation of nano-ferrofluid as sensing materials has been only limited to DC magnetic field sensing, often citing their long response time as a limiting factor to AC field sensing. This work reports the finding of high frequency (up to 15 kHz) AC magnetic field sensing capability of nanomagnetic fluid as the cladding material of a fiber-optic multimode interferometry (MMI) structure optimized for the fourth self-imaging spectral response. The key parameter enabling high frequency response is the short response time (<1 ms) achieved by optimizing both the sensing structure and nano-ferrofluid solution. Focus has been imparted on 60 Hz line-frequency profiles of various current/magnetic fields to test the efficacy of these sensors in metering and monitoring current and current-induced magnetic fields in the electrical power grid systems. The magnetic field sensitivity of 240 mV/Gauss per dBm of transmitted power was achieved for 60 Hz field applied via Helmholtz coil, whereas the 60 Hz AC current sensitivity of 2.83 mV/A was measured due to magnetic field induced by current in a straight conducting wire.

## 1 Introduction

Optical fiber based electromagnetic field sensing is a rapidly expanding field in fiber sensor technology, offering diverse sensing architectures (e.g., gratings, interferometers, specialty fibers), functional materials (e.g., magneto-optical, magnetostrictive, and diamond NV centers–quantum materials) and interrogation methods (shift in intensity, resonance wavelength, polarization, and phase) for application specific need of bandwidth, sensitivity, and range.^1−4^ The application expands from biomedical instruments,^5^ space and navigation,^6,7^ geomagnetism,^8^ electric power grid monitoring,^9^ and quantum applications,^10^ among others. Immunity to electromagnetic interference (EMI) due to dielectric silica fiber materials, compactness, low size and weight, and compatibility for remote and distributed sensing modalities are some of the advantages touted over their electronic counterpart current transducers.

With the advancement in nanomaterial synthesis and their functionalization, magnetic fluids (MFs) are one of the widely investigated and used functional materials in applications ranging from biosensing, hyperthermia treatments for cancer therapy, magnetic resonance imaging, and fiber-optic-based magnetic field sensing.^11−14^ Magnetic fluids, also known as ferrofluids, are colloidal suspensions of spontaneously magnetized single-domain nanoparticles of ferromagnetic materials, typically magnetite (Fe_3_O_4_) and maghemite (γ-Fe_3_O_4_) uniformly dispersed in a nonmagnetic carrier liquid (water, kerosene) with the help of thin layer coating of surfactant (oleic acid, ethylene glycol, and other polymers).^15,16^ When the diameter of ferromagnetic nanoparticles is below the “critical diameter” (5–15 nm), the magnetic nanoparticles (MNPs) in ferrofluid exhibit superparamagnetic behaviour characterized with zero coercivity, remanence, and hysteresis loss.^17^ The surfactant and the Brownian motion keep the homogeneous dispersion of nanoparticles stable against particle sedimentation and agglomeration. The magnetite MNPs are synthesized most commonly via chemical coprecipitation method^18−20^ for its simplicity and high yield, where ferrous (Fe^2+^) and ferric (Fe^3+^) iron salts undergo chemical reaction in basic (OH^–^) aqueous solution as
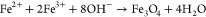
The shape, size, and phases of the nanoparticles are affected by the reaction temperature and the pH value of the basic solution.^21^

Owing to their customizable and tunable magneto-optical properties such as tunable refractive index,^22,23^ birefringence,^24,25^ and transmittance,^26,27^ the MF are popular sensing materials used in fiber-optic-based magnetic field sensing, however, largely limited to only DC magnetic field sensing, often citing their long response time as a barrier to AC field sensing.^28^ This limitation contrasts with the well- established high frequency (MHz–GHz) response observed in magnetic-relaxation spectroscopy (magnetic AC susceptibility measurements).^29^ Understanding the magneto-optical response of nano-ferrofluid at higher frequencies requires an analysis of magnetization dynamics, the two dominant relaxation processes: Neel and Brownian relaxation, and their corresponding relaxation time scales.

In the absence of a magnetic field, the magnetic moments of the particles are randomly oriented, giving rise to zero net magnetization of the nano-ferrofluid. Under a magnetic field, the particles align themselves along the direction of the magnetic field, reaching saturation magnetization at sufficiently high field strength when all of the particles are completely aligned. Assuming monodispersed particles (particles of one size) and no particle–particle magnetic interaction, the equilibrium magnetization (*M*) of the nano-ferrofluid can be descrived by the Langevin function *L*(*H*,*T*) for any applied field *H* and at temperature *T*(30) as
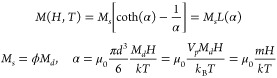
1where *M*_*s*_, ϕ, *M*_*d*_, *d*, *V*_*p*_, *m*, and *k*_B_ are saturation magnetization, volume fraction of magnetic particles in nano-ferrofluid, domain saturation magnetization of the magnetic nanoparticles (MNPs), diameter of MNPs, core magnetic volume of each MNP, and Boltzmann’s constant, respectively. For a large value of α at high magnetic field strength, *L*(α) = 1 and the MF reaches saturation of magnetization which is proportional to the volume fraction/concentration of the MNPs in the colloidal solution and the domain saturation magnetization which is higher for the larger particle size.

Once the strength of the magnetic field applied is changed, the nano-ferrofluid achieves its equilibrium by two relaxation mechanisms: First, by reorientation of the magnetic moment within the MNPs along the direction of applied field (Neel relaxation), and second, by physical rotation of the magnetic particles within the carrier liquid with internal magnetization locked to the easy axis of crystalline lattice (Brownian relaxation). Unless the particles are embedded in high viscous medium such as in a frozen liquid or in a polymer composite such as in polymethyl methacrylate (PMMA) matrix, in which case the Brownian relaxation seizes and only Neel relaxation occurs,^31,32^ otherwise, the relaxation is the combination of both mechanisms and the effective zero field relaxation time (i.e once the field is switched off) τ_*eff*_ is given as^33,34^
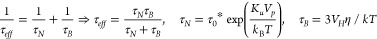
2where τ_*N*_ and τ_*B*_ are Neel and Brownian relaxation times, τ_0_ ≈ 10^–9^ s is the characteristic relaxation time, *K*_*u*_ is a monocrystalline anisotropy constant, *V*_*p*_ and *V*_*H*_ are nanoparticle’s core magnetic volume and hydrodynamic volume (*V*_*H*_ > *V*_*p*_ ≈ 3*V*_*p*_ as *V*_*H*_ includes a surfactant layer coating used for colloidal stability) and η is the viscosity of the carrier fluid. The effective relaxation time is determined by the shorter of the two relaxation times for a particular size of the particles. The Neel relaxation time contributes more to the effective time for smaller size particles, whereas the Brownian relaxation does for larger diameter particles and their agglomerated clusters.^34^ The typical value of the τ_*eff*_ is in the order of 10^–9^–10^–6^ s for MNPs of diameter less than ∼20 nm.^34^ However, in the presence of AC driving field where the magnitude of the field strength oscillates, the relaxation time is both the frequency and magnitude of field dependent. Brownian relaxation is dominant (meaning shorter relaxation time) at lower frequencies and lower magnitude of field strength, whereas Neel relaxation dominates the relaxation at higher frequencies and higher field strength.^32,33^ To respond to the AC frequency and field strength, the relaxation time should be shorter than the time period of the driving AC field.

For optical sensing of the magnetic field, magnetic fluid/nano-ferrofluid is a low-cost material that offers tunable magneto-optical properties such as refractive index (RI), absorption coefficient, and birefringence. When the magnetic field is applied, beyond a certain critical field strength, dispersed magnetic nanoparticles begin to agglomerate and form magnetic chain/column structures along the magnetic field direction until saturation.^24,35^ Dispersion and chain formation are directly related to the magnetization dynamics of nano-ferrofluid, as are the optical effects when the light interacts with the nano-ferrofluid. The magnetic field (*H*) dependent magneto-optical effects result in a tunable absorption coefficient and refractive index of the MF medium which is exploited for optical sensing of the magnetic field.

The RI of the MF, *n*_*MF*_ can be represented by similar Langevin’s equation for superparamagnetism,^23^
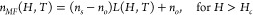
3where  is the Langevin function.

Here, *H* and *T* are applied magnetic field (Gauss) and temperature (Kelvin) of MF, *H*_*c*_ is the critical field strength (minimum field required to align the magnetic domains along the direction of field overcoming the fluid thermodynamics of the MF, below which there is no substantial chaining effect and change in RI), *n*_*s*_ and *n*_*o*_ are the RIs at saturation field (*H* = *H*_*sat*._) and at field below critical field (*H* < *H*_*c*_), respectively, and α is a fitting parameter. Besides magnitudes of *H and T*, the *n*_*MF*_ change also depends on the relative direction of the magnetic field with respect to light propagation direction (*k*), concentration of the magnetic particles in the colloidal solution, type of carrier liquid, and the light-MF interaction length.^22,23^*n*_*MF*_ increases with magnetic field intensity above *H*_*c*_, when the magnetic field is parallel to the light propagation direction (i.e., *H*||*k*) and decreases with field intensity when the H-field direction is perpendicular to the propagation direction *k* (i.e., *H*⊥*k*).^22,36^ Moreover, magnetic field has a higher refractive index modulation range for the case *H*||*k* than for *H*⊥*k*. This *n*_*MF*_ dependency on relative orientation between *H* and *k* is associated with the so-called magnetoelectric directive effect, where electric susceptibility χ change is a function of magnetic field χ = χ(*H*)^37^ and is given as
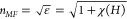
4when *H*||*k*, the electric susceptibility χ increases with *H*, i.e., , thus *n*_*MF*_ increases with *H*. When *H*⊥*k*, the electric susceptibility χ decreases with *H*, i.e., , thus *n*_*MF*_ decreases with *H*.

The tunable transmittance of MF is attributed to the microstructural changes of MF into column formation that alters the effective concentration, absorption, and scattering cross sections in the presence of increased external magnetic field.^26,38^ At zero H-fields, the MF with uniformly dispersed MNPs is essentially a liquid phase which is semitransparent to light. As the H-field intensity is increased, gradual evolution of MNPs into column-like microstructures produces a liquid–solid phase. As a result, the area cross-section occupied by the liquid phase as well as the effective concentration is reduced, as is the transparency of light. Considering these factors, transmittance *T* of light through the MF can be represented as^26^
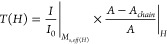
5where  is the transmittance under effective concentration *M*_*s*, *eff*(*H*)_ and (*A* – *A*_*chain*_)/*A* is the area ratio factor of the liquid phase under a given H-field given that *A*_*chain*_ is the area cross-section of the magnetic chains/columns in a total area *A* occupied by the magnetic fluid.

In this work, the sinusoidal change in optical intensity as a function of AC magnetic field at various frequencies and field strengths has been explored with particular focus on detecting current anomalies in power grid systems at 60 Hz line-frequency by exploiting multimode interferometric fiber-optic structure with magnetic fluid as the cladding/sensing material.

## 2 Experimental Methods

### 2.1 MMI Sensing Structure Optimization for Fourth Self-Imaging

**Figure 1:**
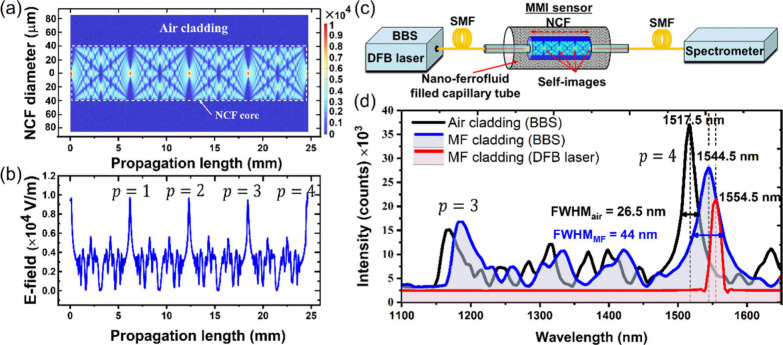
MMI fiber structure optimized for fourth self-imaging condition for a NCF of Ø(diameter) = 80 μm, *L* = 24.4 mm, and λ = 1517 nm: (a) COMSOL simulation showing interference E-field patterns and first four self-images along propagation NCF length, (b) E-field amplitude along the propagation length, (c) schematic of the MMI sensor setup for the measurement of transmitted spectrum, and (d) transmitted spectrum of the MMI sensor in air and MF cladding

Due to ease of fabrication and high sensitivity, the fiber-optic (FO) sensor based on MMI architecture has been widely utilized for multiparameter sensing, including refractive index, temperature, vibration/acoustics, strain, DC magnetic field, etc.^39^ The same interferometric architecture is adapted in this work as the basis of the sensing structure. The sensor can be easily fabricated by fusion splicing a multimode fiber (MMF) with stripped-cladding or a no-core fiber (NCF) section to two single mode fibers (SMFs) as lead fibers. The magnetic fluid is enclosed as a cladding of the MMF section inside a capillary tube, whose ends are closed with UV-curable glue. Considering circularly symmetric fiber structures and no lateral offset between the SMFs and MMF axes after fusion splicing, the single mode input from SMF excites the multiple linearly polarized LP_0m_ higher order fiber modes in the MMF. Over the length of MMF, the multiple modes propagating with different phases and velocities interfere within MMF and the modulated transmission optical response at the end of the MMF is coupled to the output SMF. The transmittance (T) from the SMS sensor structure can be given as^40^
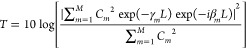
6where *C*_*m*_ is excitation coefficients of *m*th excited modes (*m* = 1, 2, 3, ..., *n*), γ_*m*_ is evanescent field absorption coefficient, and β_*m*_ is propagation constant of the *m*th order mode. When the multiple modes are excited within the multimode section of the fiber, the input field is replicated in periodic intervals along the length, also known as “self-images”. The length of the MMF section needed to create the periodic image of the input field is given as^41^
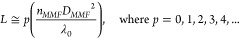
7Here *n*_*MMF*_ and *D*_*MMF*_ denote RI and diameter of the MMF core (*D*_*MMF*_ ≫ λ_0_, free space wavelength). In particular, every fourth self-image (*p* = 4*N*, with *N* = 1, 2, 3, ...) replicates the exact profile of the input field with narrow transmitted peak at a particular central wavelength. As can be inferred from eq 7, the fourth self-imaging length is longer for larger diameter fibers (*L* ∝ *D*_*MMF*_^2^)for a specific wavelength and is shorter at longer wavelengths  for a specific fiber diameter and vice versa. The simulated (beam envelope method in COMSOL 6.1 wave optics-module) electric field distribution and the corresponding transmitted intensity along the length of the multimode section of the fibers are shown in Figure 1a,b for a MMI sensor with NCF section of 80 μm diameter (NCF: Ø = 80 μm, *L* = 24.4 mm, λ = 1517 nm, *n*_*core*_ = 1.44 for silica, and *n*_*clad*_ = 1 for air cladding). The sensor is optimized to operate at a 1550 nm DFB-laser wavelength, the same as used in long distance fiber-optic communication. The details of the optimization method of the fourth-self-imaging peak to fall at a particular central wavelength is presented in authors previous work.^42^ The primary idea is to tune the sensor’s transmission spectrum so that its fourth-self-imaging peak coincides with the desired wavelength in the optical fiber communication band for maximum sensitivity or at least the full-width at half maximum (FWHM) covers the desired band of the interrogation wavelengths of laser light signal.

The optimized sensor fabrication takes a two step-process. First, a reference sensor with the same NCF core diameter is needed to quantify the peak wavelength shift when the outer medium surrounding the NCF is changed from air to an MF solution. Then the actual sensor is fabricated by accurately controlling and fusion splicing the NCF length so that the peak wavelength in air-cladding is compensated by the same amount as the wavelength shift introduced by MF cladding. A setup as shown in Figure 1c with Oceanview NIR spectrometer and a broadband source (BBS) was used to record the transmitted spectra through an SMS sensor. The fourth-self-imaging peak in air cladding was 1517.5 nm, which shifts to 1544.5 nm peak once filled with 33% DI water-diluted nano-ferrofluid (PBG-900, FerroTec Inc. USA). The FWHM broadens to ∼44 nm once filled with MF solution from its ∼26.5 nm linewidth in air as cladding. The broad FWHM of the fourth self-imaging peak is the key in making the sensor operate for a wide-band of telecommunication window. Ideally, the fourth self-imaging peak overlapped with the DFB laser wavelength is desired for highest sensitivity. However, the control in NCF length, even with the normal microscope setup, is challenging given ∼65 nm wavelength shift per mm length (i.e., Δλ/Δ*L* = 65 nm/mm) variation in an 80 μm diameter NCF section. The measurement uncertainty in length of ±100 μm will introduce ±6.5 nm peak wavelength shift from the desired 1550 nm wavelength peak (see Figure 1d). Nevertheless, the sensor would still work with prorated sensitivity, depending on the degree of deviation of the DFB laser peak-wavelength from the fourth self-imaging peak.

### 2.2 Interrogation Method and Mechanism of AC Field Sensing

**Figure 2:**
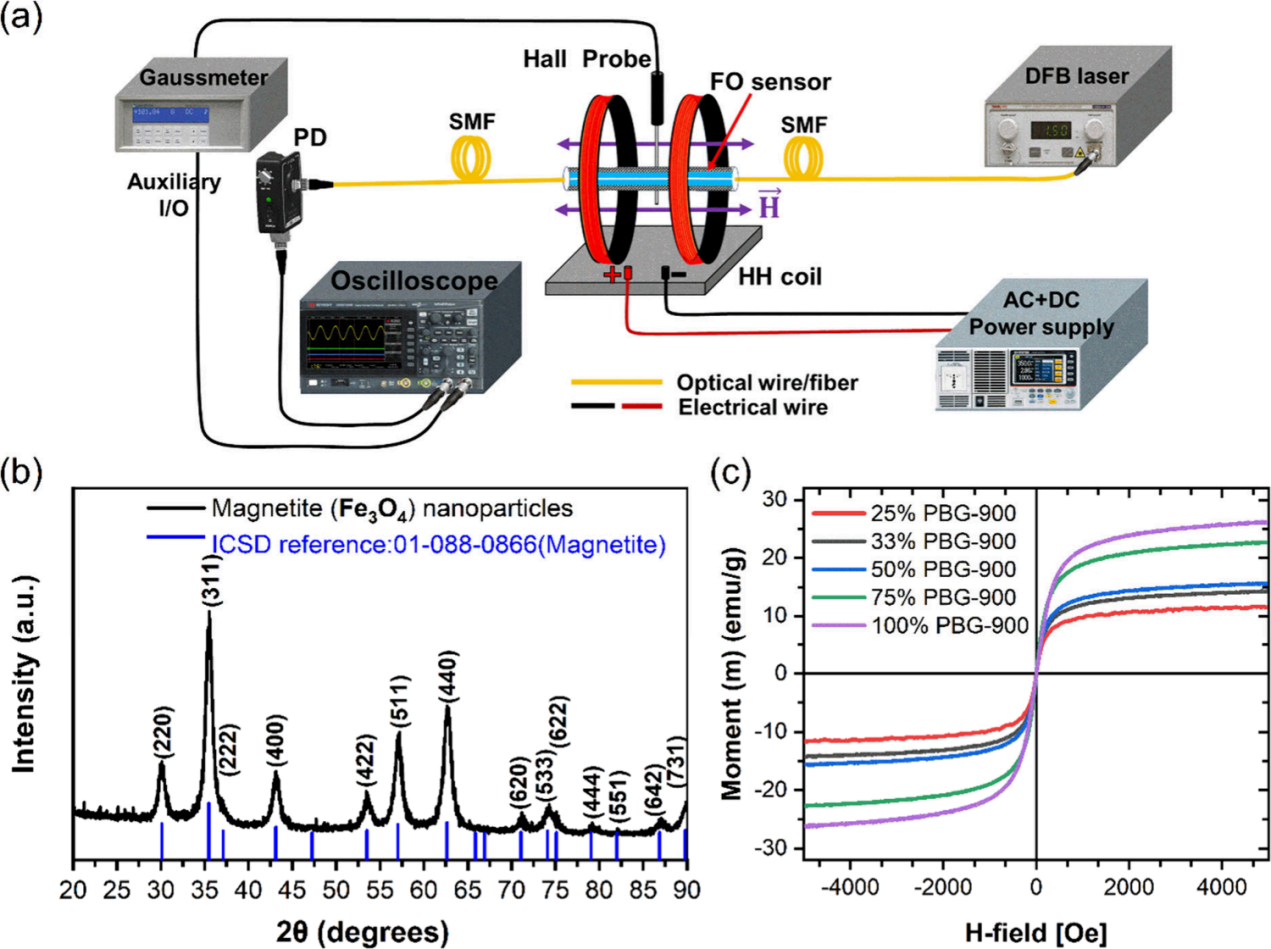
Nanoferrofluid-cladded optical-fiber sensor for AC H-field sensing: (a) interrogation setup, (b) XRD spectrum of magnetite nanoparticles, and (c) VSM measurement of M–H curves of nano-ferrofluids upon dilution.

The interrogation setup as shown in Figure 2 is used to investigate the dynamic response of the sensor subjected to time varying AC H-field excitations at various frequencies and amplitudes. The narrow linewidth optical signal with central wavelength 1554.5 ± 0.02 nm from a DFB laser (Thorlabs, S3FC1550) is transmitted through the MMI-based fiber optic (FO) sensor and is converted into an electrical signal through an InGaAs switchable gain amplified photodetector (PD) (Thorlabs, PDA10CS2). The electrical signal is then analyzed using a multichannel digital oscilloscope (Keysight, DSOX1204G, 200 MHz bandwidth, 2GSa/s sample rate). AC magnetic field is applied to the sensor parallel to sensor length using a Helmholtz (HH) coil (MH-6, Lakeshore, Max. current 2 A) which can produce a uniform magnetic field up to 55 G with a responsivity of 27.5 G/A. The current to the HH coil is supplied through an AC+DC power supply source (Instek, 2100R). In situ magnetic field is measured via a Hall probe and a gaussmeter (Model 425, LakeShore). The electrical analog signal from the gaussmeter, also connected to the oscilloscope channel, is utilized as the reference sensor signal. Synchronization of two signals utilizing the “trigger” function in the oscilloscope helps to analyze relative evolution of the dynamic signals and the sensor’s response to the AC field.

### 2.3 Role of Magnetization of Nanoferrofluids in Sensing

The intensity of the transmitted optical signal varies as a result of field dependent absorption which is a combined effect of both RI change and degree of scattering loss when the evanescent field interacts with the nano-ferrofluid,^43^ as detailed in the Introduction. The transmitted intensity decreases under magnetization at higher fields for lower concentration of MNPs, whereas it increases for nano-ferrofluid with higher MNP concentrations. These magnetic field-induced changes in the optical signal also depend on the composition of the magnetic nanoparticles.^16^ The most commonly used magnetic nanoparticles are those of magnetite (Fe_3_O_4_) for their well-developed synthesis method, also utilized in this work and verified through the XRD spectrum of Figure 2b.

The magnetic moment (M) versus magnetizing field (H) measurements for various levels of DI-water-diluted MF (PBG-900, M_sat._ ∼ 990 Oe) demonstrates higher saturation moment for MF with higher concentration of MNPs (see vibrating sample magnetometry (VSM) measurement of M–H curves of Figure 2c). The steeper slope for higher concentration of MNPs indicates that MF with high saturation magnetization is preferred for higher sensitivity (sensor's response to per unit change in the magnetic field). However, very high absorption at higher MNPs volume would yield very low transmitted photointensity and photocurrent; therefore, the preparation of optimized nano-ferrofluid solution for optical sensing is a trade-off between transmitted intensity and sensitivity. Nevertheless, a higher concentration of MNPs can be incorporated either by using a higher power laser or by amplifying the transmitted signal using an amplified photodetector. The nano-ferrofluid prepared in this work is a 33% deionized (DI) water-diluted ferrofluid (PBG-900, FerroTec Inc.), where the original 17.9% MNPs concentration by volume is lowered to ∼5.97%. The commercial nano-ferrofluid product is a water-miscible colloidal solution of polyethylene glycol (PEG)–methoxysilane surfactant-coated Fe_3_O_4_ nanoparticles (nominal particle diameter ∼ 10 nm) dispersed in DI water as the carrier liquid.

## 3 Results and Dicussion

### 3.1 Sensor Response to Square-Wave Type of Magnetic Field

**Figure 3:**
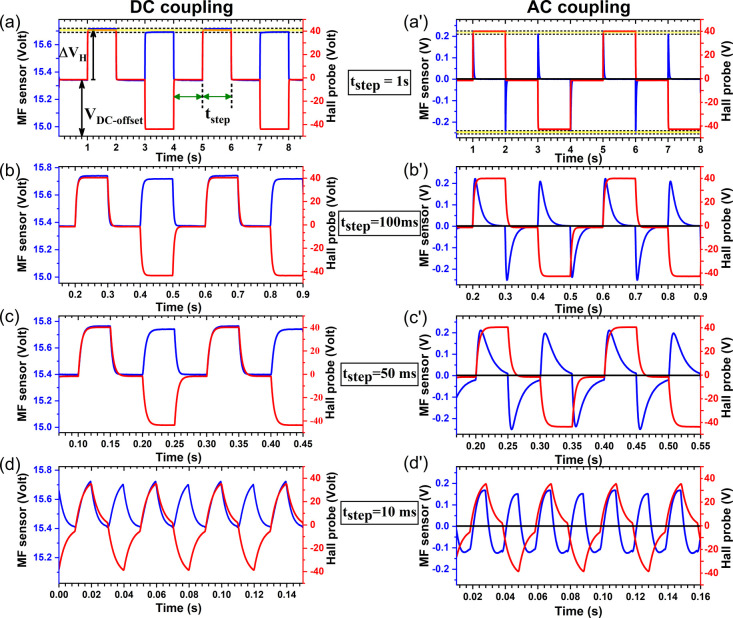
MF sensors response to pulse (ON and OFF) H-field with alternating polarity in DC coupling mode (a–d) and AC coupling mode (a′–d′) for various time steps. (Note: A DC coupling mode keeps both DC and AC components of the signal, whereas an AC coupling mode filters out the DC component and is helpful in isolating and analyzing the AC component.)

To understand the sensing mechanism and the response time of the nano-ferrofluid-cladded FO sensor, the square-wave type of pulse magnetic field with alternating polarity is applied along the sensor length through a HH coil setup. Each ON–OFF duration of H-field is reduced from 1 s to 10 ms and corresponding sensor's response is analyzed in both DC coupling mode (Figure 3a–d) and AC coupling mode (Figure 3a′–d′). The response time (rise time) of the MF sensor are measured to be ∼1 ms.

When the magnetic field is switched on, the transmitted intensity increases proportionally to the amplitude of the applied H-field and so does the photovoltage/current reading on the oscilloscope. The transmitted photointensity and the corresponding photovoltage increases even when the polarity/direction of the magnetic field is reversed, indicating that the sensor produces full-wave rectifier-circuit-like response without any discrimination against the reversal of direction of magnetic field.^44^ However, it is noteworthy that the amplitude of the MF sensor’s response differs slightly (∼0.02 V) for opposite polarities (see the yellow-colored region between two horizontal dashed-lines in Figure 3a,a′), indicating that the increase in transmitted intensity minutely differs with the direction of magnetic field. This can be attributed to the nonreciprocal effect of magnetic oxides where the propagated optical modes have a slightly different mode index and thereby different transmitted intensity when the propagation direction is parallel versus antiparallel to the magnetization direction.^45^

The AC component of the signal for every rise and fall of the pulse signal is revealed in the AC coupling mode, which is buried in the DC-offset voltage in DC coupling mode as the AC ripple is only ∼0.23 V. The short-pulse AC signal quickly rises to its maximum amplitude proportional to the applied field intensity and decays to its zero-field level. The rise time (time interval for rising edge of sensor signal to transition from 10 to 90% of the peak value) and the decay time (time interval to decay to 1/e ∼ 36.78% of the maximum value) of the AC signal are measured to be 5 and 17 ms, respectively. As the pulse duration is reduced (see Figure 3b′–d′), the sensor’s AC response does not have enough time to completely decay to its zero-field level before another rising or falling edge of the pulse signal kicks in. This is the case when the relaxation time of the MF is longer than the time scale of the driving H-field pulse. As a result, the amplitude of the MF sensor’s AC response reduces for narrow pulses.^33^ Moreover, the transient response of the sensor is on the opposite side of the zero-voltage line for the rising and falling edge of the Hall-probe signal irrespective of polarity (positive or negative value) of the magnetic field.

### 3.2 Sensor Response to Triangular Ladder-Wave of Magnetic Field

**Figure 4:**
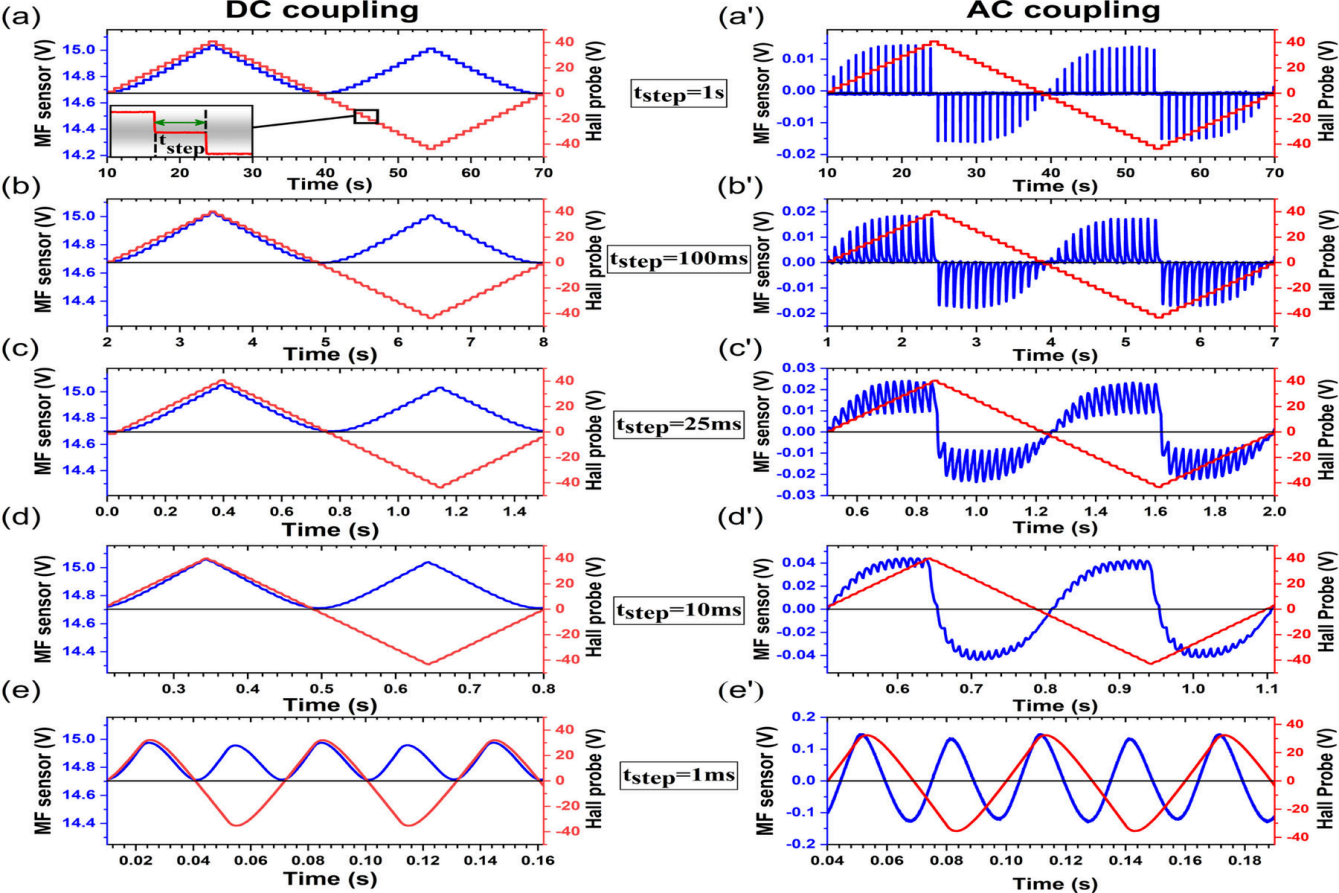
MF sensor’s response to pulse (ON and OFF) H-field with alternating polarity in DC coupling mode (a–e) and AC coupling mode (a′–e′) for various time steps.

With the foundational understanding of sensor’s response to pulse H-field response, further investigation was carried out by applying the ladder type triangular wave of H-field with its magnitude increased in discrete steps equivalent to 1 V Hall-probe signal. The ultimate aim here is to understand the mechanism of sensors’ response to a sinusoidal field where the magnitude of the field increases and decreases continuously in infinitesimal steps. As the time interval of discrete steps is reduced to 1 ms, one can see the gradual evolution of both the Hall-probe signal and the MF sensor response into a sinusoidal shape of the AC field. The signals were analyzed to see the evolution of the sensors response as the time steps were narrowed from 1 s to 1 ms in DC coupling mode (Figure 4a–e) and AC coupling mode (Figure 4a′–e′). The MF sensor replicates the exact profile of the input H-field as represented by the Hall-probe signal, except for the indiscriminate response to both polarities and a slight difference in amplitude for opposite polarity of H-field due to nonreciprocal magneto-optic effect as discussed above.

In the AC coupling mode, for the time steps longer (>∼100 ms) than the relaxation time of MF sensor's response, the transient response for each step starts at the zero-voltage reference line and increases with increased H-field. However, upon reducing the time steps to 50 ms or less, the transient response builds upon the response to previous steps as the MF does not completely decay down/relax to zero magnetization state. In other words, the net amplitude is a cumulative effect of sensors’ response to incremental discrete steps. For example, the ±0.15 V peak-voltage of MF sensor response for 1 ms interval steps is about 10 times larger than the ±0.015 V peak-voltage for 1 s interval steps. This indicates that the MF sensor is more sensitive at higher frequency AC H-field (lower time interval steps). It should also be noted that for each half-cycle of the triangular input signal, the MF sensor response completes a full cycle as the sensor response switches direction for each rising and falling side of the edge. This explains why the MF sensor response signal is double in frequency of the input signal.

### 3.3 Sensing Sinusoidal AC H-field of Various Frequencies and Field Strengths

The sinusoidal AC signals are the most common and important signals from their practical applications standpoint. Therefore, the sensor is finally put to test against the sinusoidal current induced H-field of various strengths from 0 to 55 G and frequencies up to 15 kHz. The sensor’s response signals are analyzed against the reference signals measured by a commercial Hall probe. In particular, the harmonics components of the sensor’s response signal, when exposed to a 60 Hz line-frequency current induced H-field signals, are analyzed via the fast Fourier transform (FFT) method, and the sensitivity values are calculated at various harmonics components.

#### 3.3.1 Sensor Response to a 60 Hz Line-Frequency Current Induced Magnetic Field

**Figure 5:**
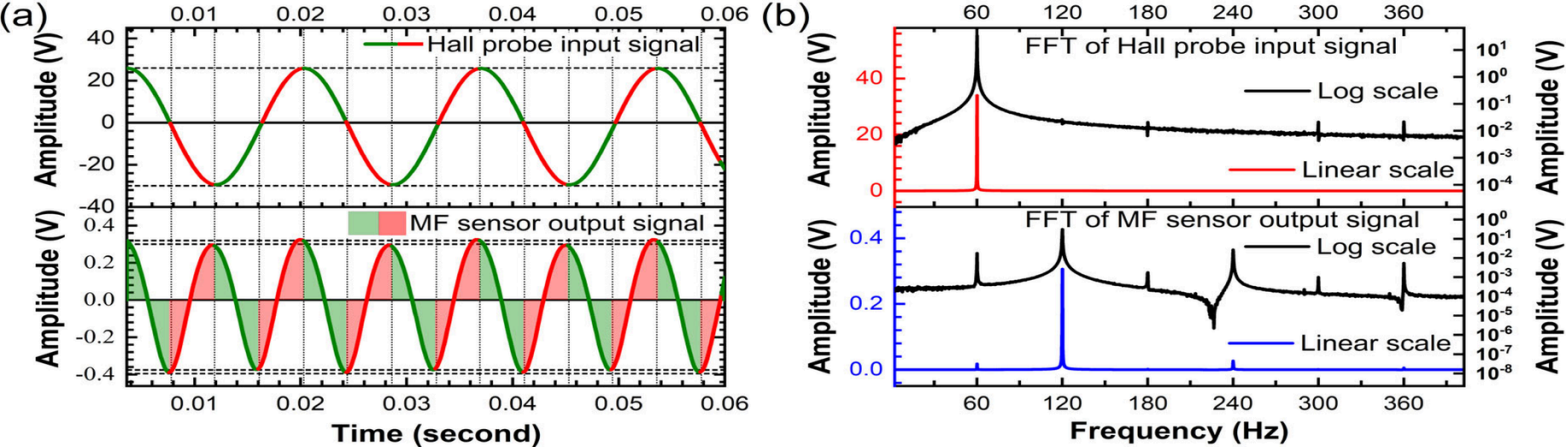
MF sensor response to 60 Hz AC H-field: (a) mechanism demonstrating response of MF sensor with reference to Hall probe signal and (b) analysis of harmonics content in the Hall probe input and MF sensor output signal.

One of the objectives of this study is to extend the sensor’s ability to measure and monitor the magnetic field due to 60 Hz frequency alternating current and their harmonics in transmission line and power grid system. The harmonics can be present in the current supply line as a result of different power generation plants integrated in a power grid system.^46,47^ The MF sensor is thus exposed to pure 60 Hz sinusoidal AC H-field supplied via the current through the HH coil, as indicated by the Hall probe signal and its FFT spectrum in Figure 5a,b. The other higher order harmonics are invisible in linear scale but are visible in the logarithmic scale. The amplitude of 60 Hz frequency component is the dominant one as evinced by fast Fourier transform (FFT) spectrum of the input signal. The MF sensor's response, however, converts 60 Hz input frequency to a 120 Hz frequency as the dominant spectrum containing about 82% of the signal’s total amplitude. This is because the AC response of the sensor reverses the direction for each falling and rising edge of the sinusoidal signal irrespective of the direction of applied H-field. Figure 5a shows the alternating green and red section of the Hall-probe signal (top) representing the rising and falling side of the signal with the corresponding MF sensor’s response (bottom) in shaded-green and shaded-red regions, respectively. The other major harmonics in the order of their average % contribution to the total signals are 240, 360, and 60 Hz with respective contributions of 9.8%, 5.3%, and 2.1% (see Table 1).

**Table 1:** Sensitivity and Response–Linearity at 60 Hz Harmonics

	Even harmonics			Odd harmonics		
60 Hz Harmonics	120 Hz	240 Hz	360 Hz	60 Hz	180 Hz	300 Hz
Av % of total signal	82	9.8	2.1	5.3	0.5	0.3
Sensitivity (mV/Gauss)^a^	9.91 ± 0.021	2.02 ± 0.015	0.52 ± 0.06	0.37 ± 0.02	0.06 ± 0.002	0.05 ± 0.002
R^2^	0.9950	0.9405	0.8734	0.9688	0.9836	0.9714

a The mV values are proportional to the transmitted photointensity through the sensor. These reported values correspond to 52.19 μW of transmitted intensity through the sensor when a 2.48 mW DFB laser was used as the input laser source at 1550 nm.

**Figure 6:**
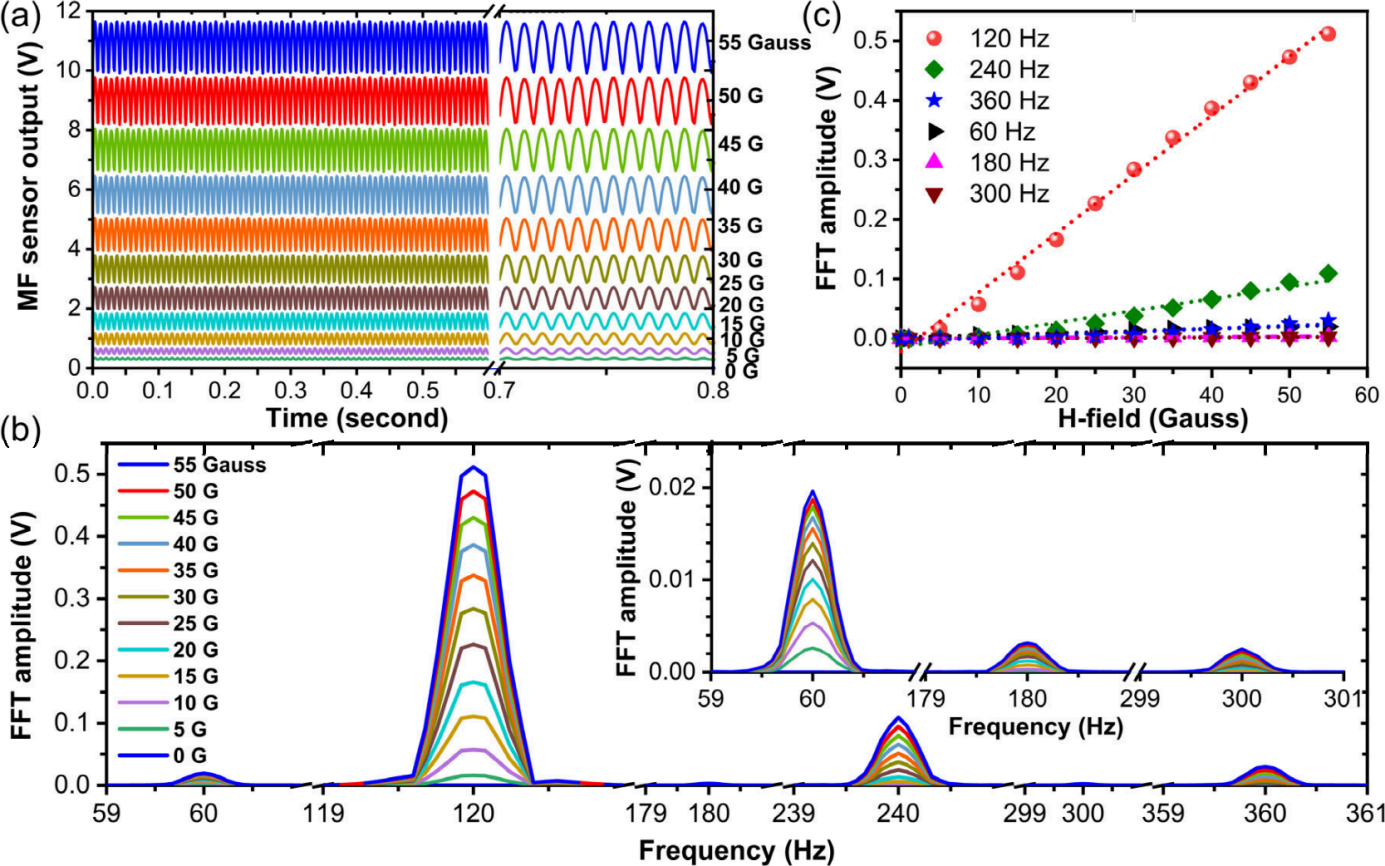
MF sensor’s response to a 60 Hz AC H-field of various strengths: (a) MF sensor's output AC signals at various strengths H-field, (b) their FFT amplitudes, and (c) linearity of FFT amplitudes with magnetic field.

The sensor responds linearly to the increased magnetic field with output signal’s *peak-to-peak voltage (V*_*pp*_) value and their FFT amplitudes increasing at higher field applied up to 55 G (see Figure 6a–c). The maximum field applied here is only limited by the HH coil’s current-limit of 2 A. The sensor is found to be most sensitive at even harmonics frequencies, and the highest sensitivity measured is 9.91 mV/Gauss for 120 Hz component with near perfect linearity (R-square value = 0.995). The highest of amplitudes among even harmonics at 120 Hz is about 25 times larger than the highest of amplitudes among odd harmonics at 60 Hz for a given magnetic field flux density. Nevertheless, the significant signal (∼5%) at 60 Hz baseline frequency makes the MF sensor useful in tracking different current/magnetic field profiles/anomalies at the same line-frequency. The performance of the sensor at different harmonics in the order of their sensitivity levels along with their respective degrees of response-linearity is tabulated in Table 1. The tabulated sensitivity values correspond to the 52.19 μW transmitted power through the sensor when 2.48 mW of input power from a DFB laser at 1550 nm was coupled to the sensor.

#### 3.3.2 Dependance of Sensitivity on Transmitted Power

**Figure 7:**
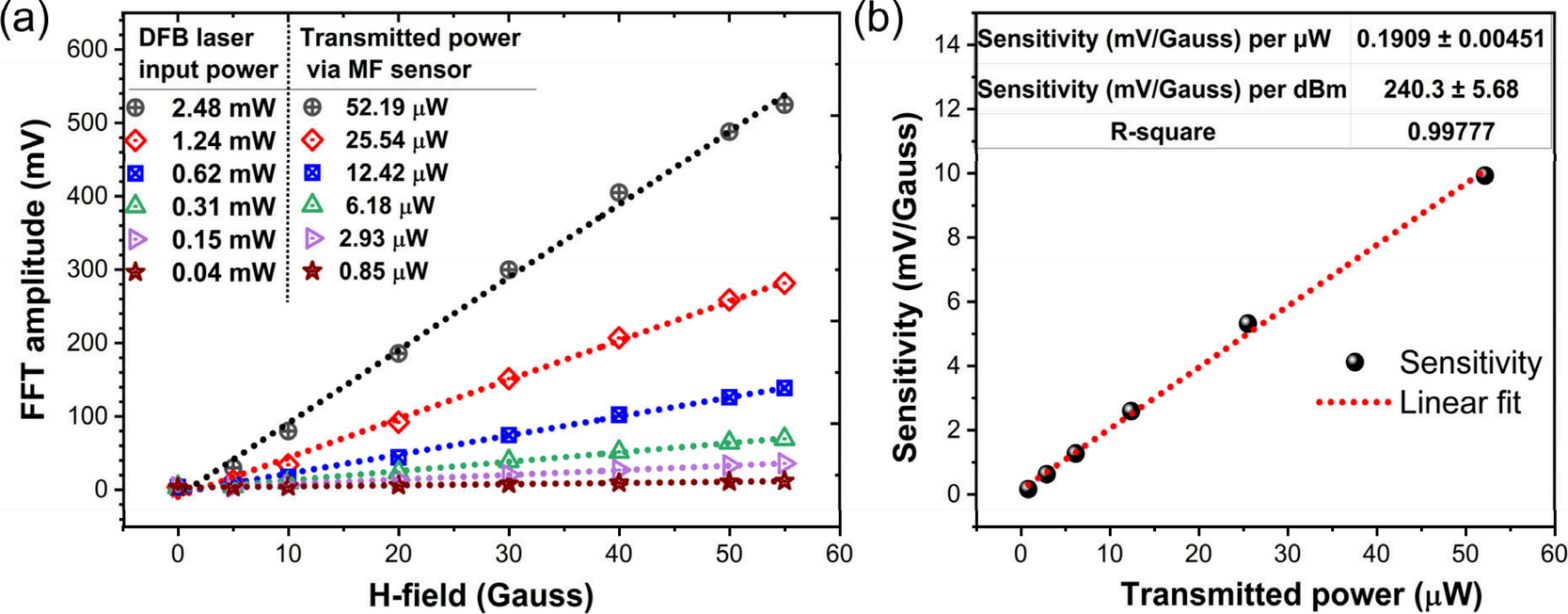
Dependance of sensitivity on transmitted power through MF sensor for a 60 Hz AC H-field: (a) FFT amplitude of sensor’s response for various input DFB laser power and transmitted power through MF sensor and (b) sensitivity at various transmitted powers through the sensor. (Note: The sensitivity values measured here correspond to FFT amplitude of 120 Hz output harmonic component for a 60 Hz input H-field signal.)

To investigate the sensitivity dependence on the amount of transmitted power through the MF sensor, the FFT amplitude of output signal at 120 Hz harmonic was recorded by varying the transmitted power through sensor upon different input laser power coupled to the input port of the fiber and H-field applied between 0 and 55 G (Figure 7a). The inset table of Figure 7a shows the transmitted power corresponding to different input laser powers with a transmittance of only ∼2.045% (−16.89 dB). The sensitivity values (slopes of the fitted curves of Figure 7a) shows ta linear dependance on the amount of transmitted power through the sensor (see Figure 7b). The sensitivity was measured to be 0.1909 ± 0.00451 mV/G per μW (equivalently, 240.3 ± 5.68 mV/Gauss per dBm) of transmitted power through the sensor. The higher the input laser power, the more light transmits through the sensor and interacts with the magnetic fluid, thereby increasing the sensitivity. Thus, the sensitivity can be further increased by reducing the optical coupling loss at the interfaces and at fusion splicing joints or by using higher power laser.

#### 3.3.3 Sensor Response to Higher Frequency Magnetic Field

**Figure 8:**
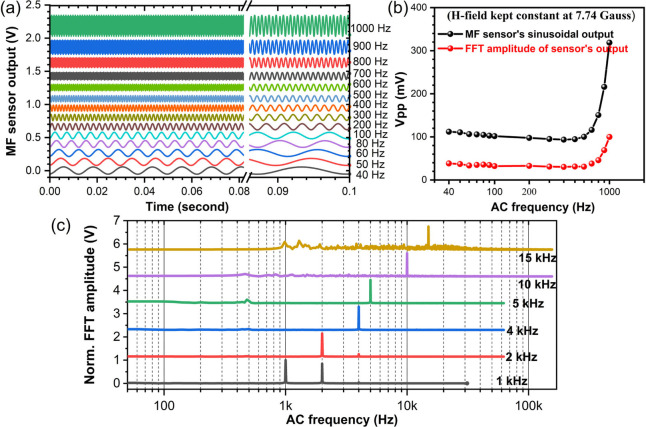
MF sensors response at higher frequency H-field: (a) sensor’s response to AC frequency up to 1 kHz at a constant H-field of 7.74 G, (b) amplitude of the sensor’s response signal, and (c) frequency response above 1 kHz H-field.

The sensor’s high frequency response from 40 Hz to 1 kHz, limited only by the AC power supply unit's capability, was also tested by keeping the current through the HH coil the same. The maximum magnetic field of 7.74 G at 1 kHz frequency for the highest possible current delivered by the power supply unit in-use is maintained constant across all lower frequencies. The stacked AC signals output from MF sensor are shown in Figure 8a. The variation of *V*_*pp*_ at higher frequencies shows that the sensitivity remains more or less the same below 700 Hz but increases rapidly above, indicating high sensitivity at high kHz frequencies (Figure 8b).

The sensor’s response at frequency beyond 1 kHz up to 15 kHz is recorded by applying the H-field via a dipole-electromagnet with which relatively higher magnetic field can be generated for smaller current at higher frequencies. The high frequency signals are generated using a combination of a waveform generator and a power amplifier. The magnetic field is however applied perpendicular to the propagation direction due to limitation posed by the geometry of the dipole-electromagnet. The sensor successfully detected frequencies up to 15 kHz as evinced by the normalized FFT amplitude plots in Figure 8c. Notably, the fundamental frequency (first harmonic) is the major component at higher frequencies above 1 kHz. The sensor is anticipated to respond to even higher frequency with further engineered MNPs in liquid or solid phase media and/or improved excitation and detection systems. The sensors capability in sensing a high-frequency current-induced AC magnetic field opens up the possibility of sensing applications in ultrafast electronic circuits and switches.

### 3.4 Electrical Power Grid Monitoring Applications

#### 3.4.1 Sensing Current in Straight Conducting Wire/Power Transmission Lines

**Figure 9:**
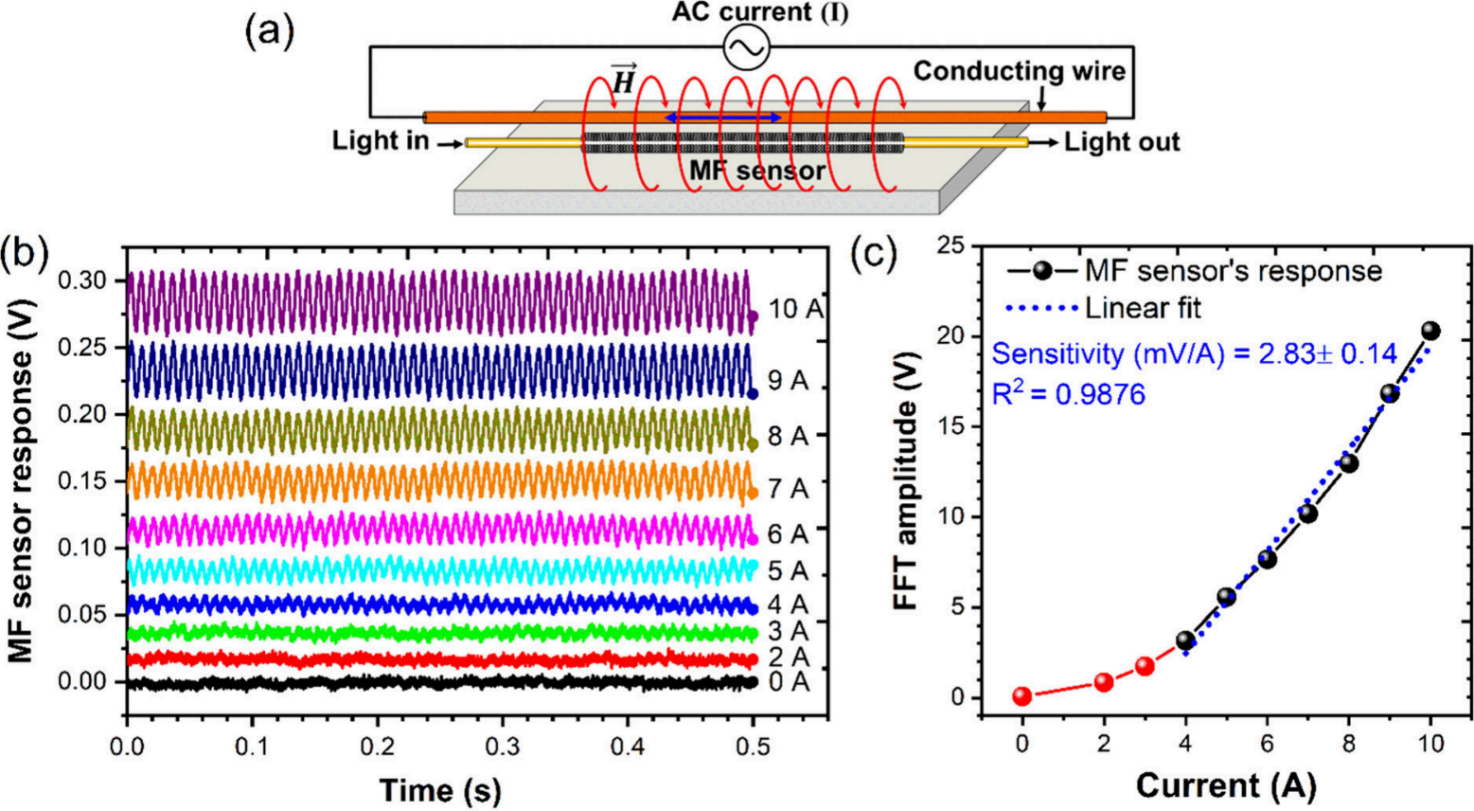
MF sensor response to 60 Hz H-field induced around a straight current carrying conducting wire: (a) schematic of the setup, (b) MF sensor's output AC signal, and (c) FFT amplitude variation with the current magnitudes.

In practical field deployment in monitoring the current in power transmission lines, the sensors must be capable of detecting the 60 Hz AC magnetic field of relatively lower magnitude compared to the field generated by the HH coil. The magnetic field induced due to current in a straight wire
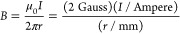
is about 2 G per ampere current in the wire at a mm distance from the center of the wire. When the sensor is deployed parallel to the wire, the magnetic field lines will encircle the wire and hence lie perpendicular to the propagation direction of the light. To test the efficacy of these sensors in detecting current in a straight conducting wire, the MF sensors is deployed about ∼2 mm distance from the straight copper wire through which the 60 Hz AC current of RMS amplitude up to 10 A is injected (Figure 9a). At a current below 2 Amp, the MF sensor’s reception signal is noisy due to low magnetic flux and low signal-to-noise ratio (SNR). However, above 3 Amp current, the FFT amplitude of sensors response signal increases linearly (Figure 9b,c) with the higher amperes of current in the wire with a good linear correlation (*R*^2^ = 0.9876) and calculated sensitivity of 2.83 ± 0.14 mV A^–1^. Thus, the same sensor also works as the current sensor, whose sensitivity can be further increased by reducing the separation of the sensor and the current wire, thereby increasing the magnetic flux. Note that the magnetic field produced by a 10 A current carrying wire at a 2 mm distance away from the axis of the wire is ∼10 G. This result demonstrates that the sensor is capable of detecting a low magnetic field. Given that the sensors have been demonstrated to respond up to 55 G field and above, applied through HH coil, the current in a straight wire to produce the same field needs to be in 100s of amperes. Moreover, the sensor can be placed further away from the center of the wire to lower the H-field induced and enabled for measuring kilo-amperes of current prevalent in electrical grids and supply lines during current fault events.

#### 3.4.2 Various Current Profiles/Faults Detection and Monitoring

Lastly, the MF sensors are investigated for their ability in detecting different current profiles that exists in different electrical circuits and components including those during short circuit current faults events in power transmission lines and other assets of power grids systems.^48−50^ Since the base supply frequency is 60 Hz, the focus has been imparted on hardware simulation to generate different current profiles at the same frequency. The current is supplied again through the HH coil, and the sensor response is recorded in terms of electrical voltage output through oscilloscope. As discussed above, for a base 60 Hz input field frequency, 120 Hz is the major frequency component in the MF sensors signal. However, the 60 Hz spectral component still possesses measurable amplitude, which can be used to extract the 60 Hz current profile embedded in the signal by filtering other frequency components.

**Figure 10:**
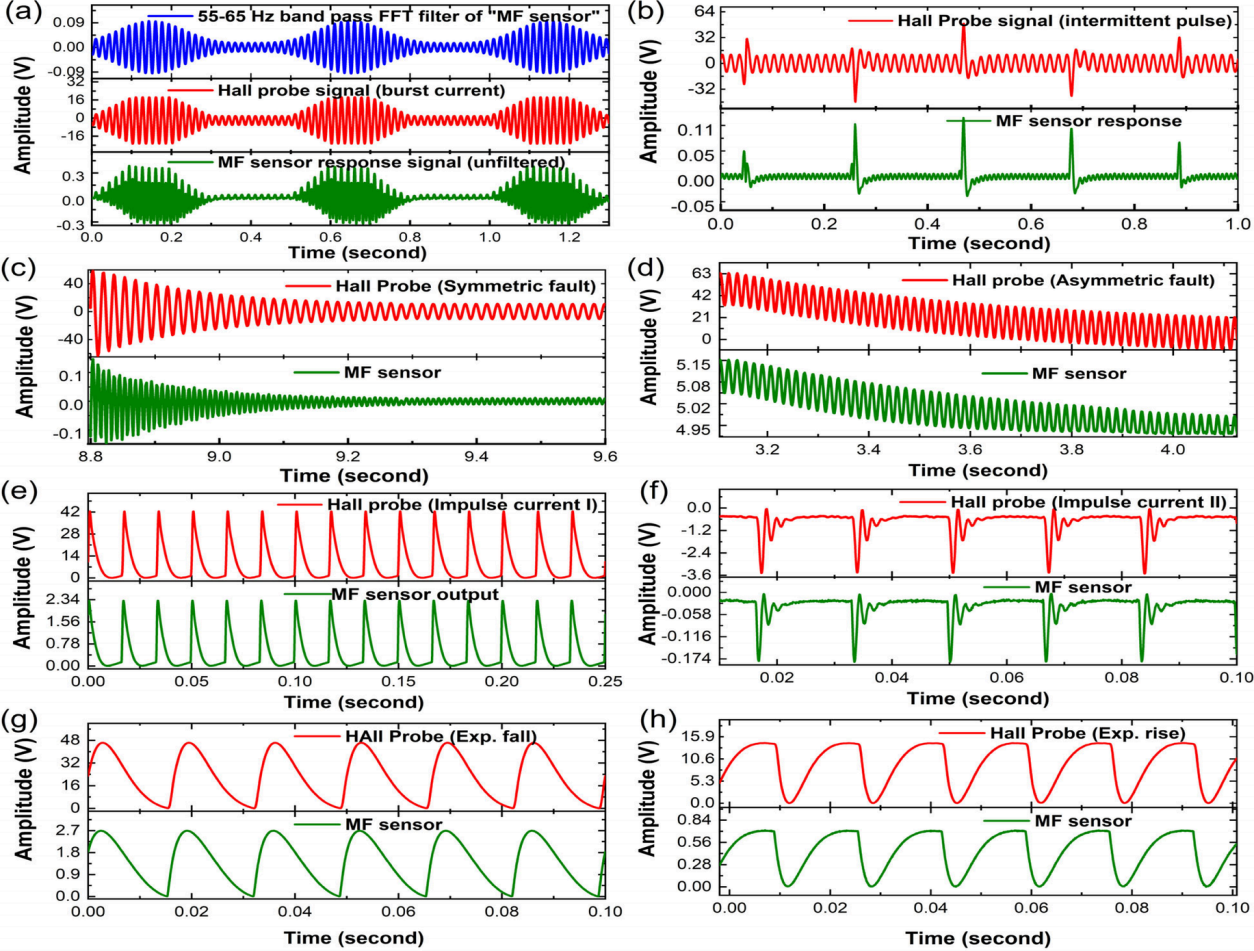
MF sensor response to several types of current profiles: (a) burst current, (b) intermittent spikes/pulse, (c, d) symmetric and asymmetric current profiles during short circuit faults, (e, f) second-order step and impulse current; (g, h) exponential fall and rise current.

For example, in Figure 10a, for the burst current profile (red color plot) fed to the HH coil and measured by the Hall probe, the MF sensor produces the response signal represented by the graph in green. After applying a narrow bandpass filter between 55 and 65 Hz to the output MF sensor signal, the filtered signal (blue color graph) can replicate the exact profile as the input current profile. The sensors thus can be used to monitor several current profiles that result from different anomalies in the circuits. Several types of current profiles are generated mimicking intermittent spike current profile (Figure 10b), symmetric and asymmetric fault current profiles (Figure 10c,d) of short-circuit events in power transmission lines, two types of impulse current profiles (Figure 10e,f), and exponential fall and rise current profiles (Figure 10g,h).The sensor replicates the exact same current/H-field profile as long as the polarity/direction of the current/H-field is in the same direction such as the current profiles of Figure 10d–h.

## 4 Conclusion

A magnetic fluid-cladded multimode interferometry-based fiber optic sensor is optimized for fourth self-imaging conditions enabling interrogation at the telecommunication C-band wavelength. The mechanism of sensor’s dynamic response has been analyzed by applying pulse H-field of various pulse widths down to 1 ms. It has been found that the optical response time of the sensor can be decreased to ∼1 ms by using the optimized nano-ferrofluid of high saturation magnetization. As a result, the sensor enables the sensing of high frequency AC H-field in the kHz range. However, the sensor responds indiscriminately to the opposite polarity of the AC field producing a full-wave rectifier like signal with double the frequency for any input AC current/H-field frequency. For example, at a power supply line frequency of 60 Hz current, 82% of the signal amplitude lies within second harmonics signal at 120 Hz, followed by ∼10 and ∼5% at 240 and 60 Hz harmonics, respectively. The maximum sensitivity of 9.91 mV/Gauss and near perfect linearity (*R*^2^ value of 0.995) was achieved at 120 Hz (second harmonics) for ∼52 μW of transmitted power through the sensor within 0–55 G range of magnetic field. As with any intensity-based interrogation of sensor, the sensitivity can be increased with higher transmitted power through the fiber sensor, which can be achieved by reducing the coupling losses at the interfaces and by using the higher input laser power. The shorter response time but longer decay time of the MF sensor’s response results in higher sensitivity at higher frequency AC input signal and vice versa.

The MF-cladded MMI sensor is able to successfully detect the AC magnetic field induced around a current carrying a straight conducting wire with a sensitivity of 2.83 mV/A and linearity (R-square) of 0.98, making this sensor compatible with deployment along the power transmission line. The sensor is also capable of replicating several types of transient current profiles at the line frequency, extending its usefulness in measuring and monitoring different current profiles and anomalies that are generated during various possible current fault events during the operation of the power grids.

## Abbreviations

AC: alternating current
DC: direct current
DFB: distributed feedback
DI water: deionized water
FFT: fast Fourier transform
fwhm: full width at half-maximum
H-field: magnetic field
MF: magnetic fluid
MF sensor: magnetic field sensor
MMF: multimode fiber
MMI: multimode interferometry
MNP: magnetic nanoparticles
NCF: no core fiber
PD: photodetector
SMF: single mode fiber
VSM: vibrating sample magnetometry
XRD: X-ray diffraction
